# Intravascular Rewarming in Major Burns: A Rare but Serious Catheter-Related Complication

**DOI:** 10.3390/ebj7010010

**Published:** 2026-02-09

**Authors:** Theodora Ligomenou, Eirini Nikolaidou, Argiro Pipinia, Georgios Trellopoulos, Stavros Galanis, Myrto Tzimou, Georgia Vasileiadou, Sophia Papadopoulou

**Affiliations:** 1 Plastic and Hand Surgery Department & Burn Unit, “G. Papanikolaou” Hospital, 57010 Thessaloniki, Greece; t.ligomenou@gmail.com (T.L.);; 2 Vascular Surgery Department, “G. Papanikolaou” Hospital, 57010 Thessaloniki, Greece; 3 Interventional Radiology Department, “G. Papanikolaou” Hospital, 57010 Thessaloniki, Greece; 4 A’ General ICU, “G. Papanikolaou” Hospital, 57010 Thessaloniki, Greece

**Keywords:** intravascular rewarming, intravascular warming device, catheter, complication, major burns, hypothermia

## Abstract

**Introduction**: Patients with major burn injuries are highly susceptible to hypothermia due to extensive skin loss, aggressive fluid resuscitation, repeated surgical procedures, and exposure during wound care. Hypothermia is associated with coagulation disorders, increased blood loss, impaired immune response, prolonged hospitalization, and increased mortality. When conventional warming strategies fail, intravascular temperature management systems may be employed, although they carry risks inherent to central venous catheters. **Case Report**: We report the case of a 26-year-old male with 66% total body surface area flame burns and inhalational injury, admitted to the Burns Intensive Care Unit with persistent hypothermia despite standard warming measures. An intravascular temperature management catheter was inserted via the femoral vein and successfully restored normothermia. Due to clinical instability, the catheter remained in situ beyond the recommended duration. During attempted catheter removal, significant resistance was encountered, raising concern for mechanical malfunction. Imaging confirmed catheter entrapment without fracture. Multidisciplinary management involving vascular surgery and interventional radiology enabled successful removal using endovascular snare techniques. A detached balloon fragment was identified and secured with venous stenting. **Conclusions**: This report describes the first documented case of complicated removal of an intravascular warming catheter due to balloon detachment in burn patients. Physicians using these devices should be aware of this possible complication and be prepared for its management.

## 1. Introduction

Patients with major burns are at greater risk of hypothermia than other surgical patients due to extensive loss of skin integrity, large-volume fluid resuscitation, hydrotherapy sessions, moist dressings, prolonged and repeated surgical procedures, anesthetic-induced thermal derangement, and mechanical ventilation, all of which contribute to heat loss [1,2,3]. Hypothermia poses a significant therapeutic challenge with deleterious effects on cutaneous blood flow (and wound healing), coagulation (increased blood loss and transfusion requirements during surgery), immune function, increased myocardial complications, increased intensive care unit (ICU) stay, prolonged hospitalization, and increased mortality [3,4,5]. Current strategies to prevent and treat hypothermia include increasing ambient temperature in the ICU and operating theatre, warming all administered and externally used fluids, and employing forced-air warming systems; however, no single intervention or combination of measures is consistently effective [6]. When conventional methods fail, advanced temperature management systems, such as intravascular or esophageal warming devices, may be employed [4]. Recently, at our institution, we used the ICY^TM^ intravascular balloon-catheter system connected to the Zoll^®^ Thermagard^TM^ device in cases where conventional warming techniques were not sufficient to keep the patient normothermic with satisfactory results. Corallo et al. first described the use of this device in 2008 in a case report, during a large burn excision with excellent results [6]. The technique, though, is invasive and carries the risks associated with central venous catheters (CVCs), as well as potential coagulation disturbances in the context of the already altered hemostatic profile of burn patients [4,7,8]. CVC-related complications, such as catheter breakage and embolism, may occur during insertion, maintenance, and removal [8]. Alternatively, esophageal warming devices offer a less invasive approach to core temperature regulation by conductive heat exchange through circulating warm water; however, there are no published clinical studies comparing the two methods regarding their safety and efficacy [4].

The following case report details a serious complication arising from application of the intravascular closed-circuit, thermostat-controlled, warm-water circulating balloon catheter for rewarming a 26-year-old male patient with a 66% total body surface area (TBSA) severe thermal burn, with persistent hypothermia. We describe the timeline of the incident that occurred during an attempt to remove the catheter, and the steps and methods undertaken by a multidisciplinary team, to successfully manage the situation encountered.

## 2. Case Report

A 26-year-old male patient sustained flame burns covering 66% of his total body surface area (TBSA), as a result of a gas cylinder explosion, while cooking in his apartment. He had no significant previous medical history. Initial resuscitation, including endotracheal intubation and fluid resuscitation based on the Parkland formula, was initiated at a district hospital prior to his transfer to our Burns Intensive Care Unit (BICU) six hours post-burn.

Upon BICU admission, a detailed assessment revealed deep partial and full-thickness burns affecting his head and neck, trunk, upper and lower extremities, while inhalational injury was confirmed by bronchoscopy. The abbreviated Burn Severity Index score (ABSI) was 11, and the revised Baux score was 109.

At admission, his core temperature was recorded at 35.5 °C. Despite all measures to keep the patient normothermic by raising the ward temperature, overhead warming red lamps, a warm air blanket, and warm intravenous fluid administration, his core temperature gradually dropped below 34 °C, especially after enzymatic debridement with associated periprocedural soaking of both lower extremities on post-burn day 1.

**Figure 1:**
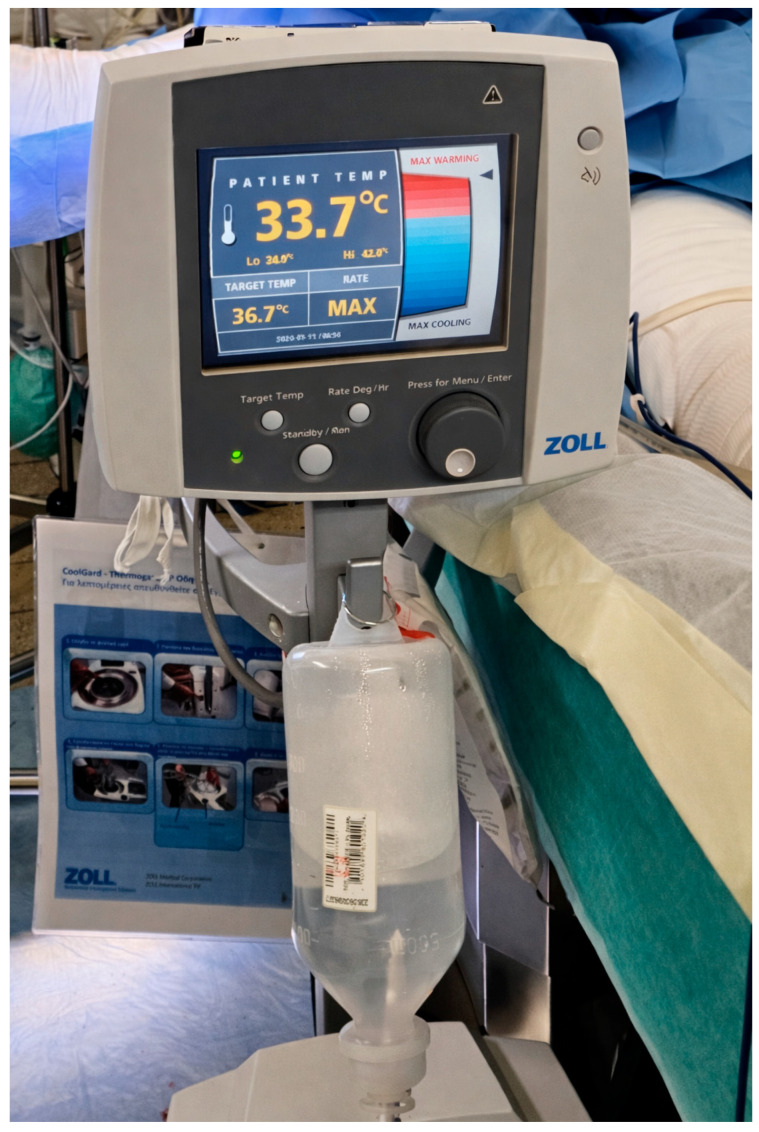
ThermogardXP^®^ Zoll^®^ console in theatre, showing patient’s core temperature of 33.7 °C.

**Figure 2:**
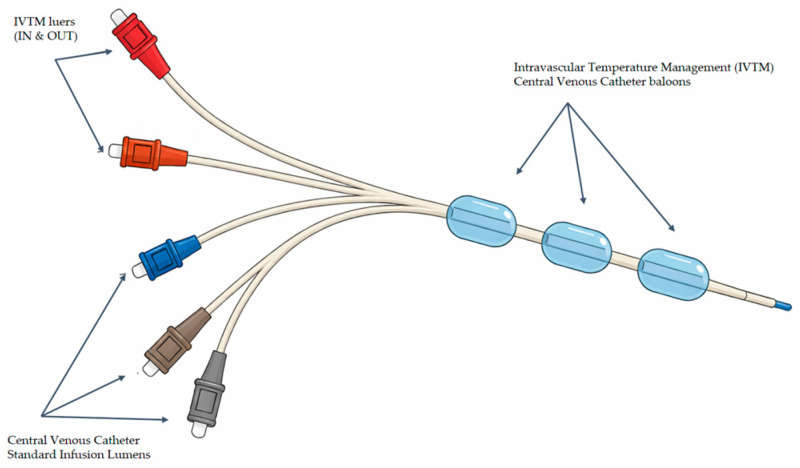
Intravascular temperature management (IVTM) central venous catheter with its components.

As he was not responding to traditional warming methods and remained hypothermic, a 38 cm ICY^TM^ Zoll^®^ intravascular temperature management (IVTM) central venous catheter (CVC) was inserted into his inferior vena cava through the right femoral vein, under sterile conditions, with the Seldinger technique, on post-burn day 2, and connected to a ThermogardXP^®^ Zoll console (Figure 1). This catheter contains a thermistor that continuously monitors the patient’s temperature and adjusts it using a plastic cold/heat-resistant multiballoon heat/cold exchange system (Figure 2). Temperature-controlled sterile saline circulates through the balloons in a closed-loop system (not infused into the patient), allowing for active warming or cooling of the circulating blood and consequently the patient. Once the set target temperature is reached, the patient is kept at this temperature. The device remains in continuous “Run Mode” until it is no longer deemed clinically necessary, at which point a test in “Standby Mode” is performed [9].

The target temperature for our patient was set at 37 °C. His core temperature gradually improved, with fluctuations and drops during surgeries. The ICY^TM^ Zoll^®^ catheter is normally validated for 4 days, as recommended per instructions for use, but since the patient was unstable and there were no suitable alternative sites for another CVC, it was decided to keep the IVTM system. It was used in the BICU ward and in theatre on and off until post-burn day 9, when surgical escharectomy of both upper extremities and grafting was performed. On post-burn day 10, with a stabilized and normothermic patient, removal of the catheter was decided. The patient was still ventilated and sedated in critical care.

But what would be a routine CVC removal procedure ended up being a nightmare. After disconnecting from the system, aspirating the remaining saline as per instructions, and cutting the holding suture, the catheter could only be pulled for a few centimetres, and then resistance was felt, as if it was “stuck”. No force or violent attempts were made; the impression was that the plastic balloon was partly detached from the shaft of the catheter and folded on itself into the vessel lumen, impeding the atraumatic and safe removal of the catheter. The X-ray performed showed that the CVC was in the right iliac vein and no part of it was broken.

**Figure 3:**
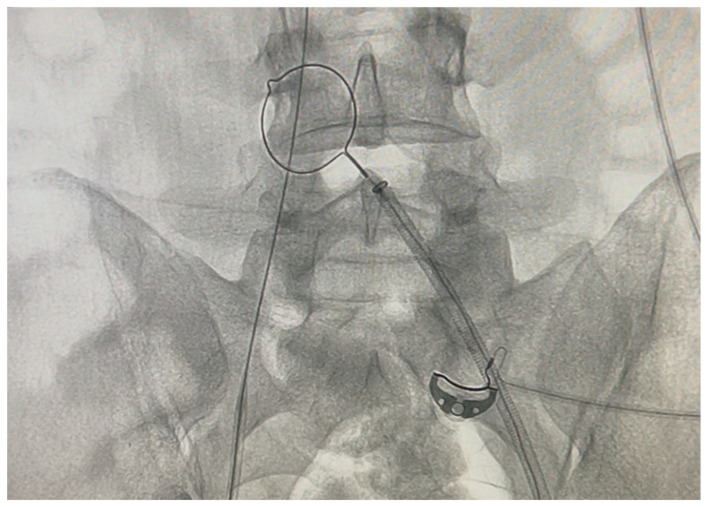
Amplatz Goose Neck^TM^ Snare inserted.

**Figure 4:**
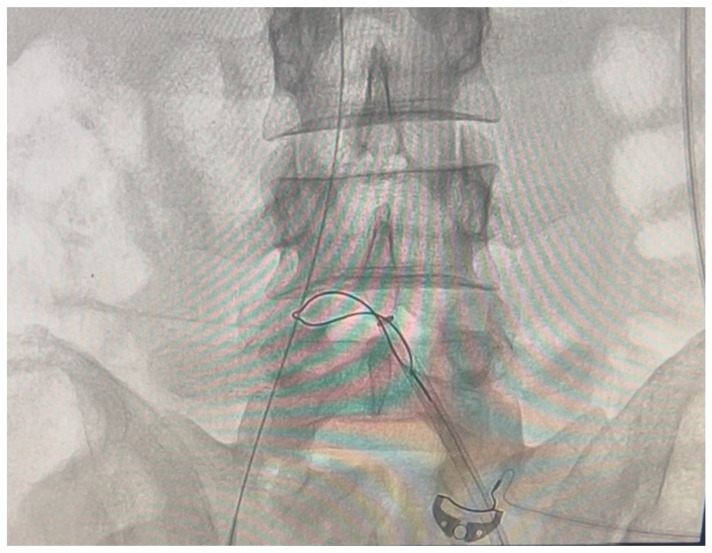
Radiography during the catheter removal with the Snare Kit.

**Figure 5:**
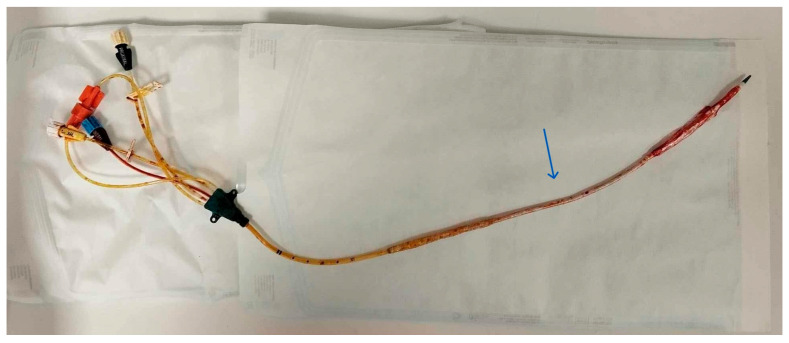
The removed ICY^TM^ stuck catheter. The middle balloon is missing (arrow).

A vascular surgery consultation was ordered immediately by the attending BICU plastic surgeon and intensivist. It was decided to transfer the patient to the Interventional Radiology department, where two interventional radiologists and the vascular surgeon attempted and accomplished the catheter removal. No findings of deep vein thrombosis or local signs of infection were evident. At first, an effort was made to retract the catheter, inserting a sheath as a dilator in the right femoral vein, without success. Under continuous angiographic visualization, catheterization of the contralateral (left) femoral vein was then performed using the Seldinger technique. A 0.035-inch soft wire was introduced as a diagnostic catheter. Once the end of the stuck catheter was located in the right iliac vein, a snare kit (Amplatz Goose Neck^TM^ Snare kit, ev3^TM^ Inc, Plymouth, MN, USA) was inserted, and a 20 mm goose-neck snare was looped around the free end of the catheter and tightened to secure it (Figure 3 and Figure 4). Attempts were made to pull the catheter retrogradely through the left femoral vein through a sheath, without success. Finally, it became possible to pull the catheter out from the right femoral entry point. During the removal, a foreign body was seen (on the angiography screen) floating, assuming it was a piece of the broken balloon. Every effort should be made to prevent it from travelling up the inferior vena cava and causing embolism. After unsuccessful attempts to catch it with the loop, it was transfixed on the vessel wall of the left external iliac vein with a venous stent. After the catheter removal, an Angio-Seal^®^ closure device was placed. On observation, the middle balloon of the catheter (ICY^TM^ has three balloons) was missing, while the shaft of the catheter was intact (Figure 5). The whole procedure in the radiology department lasted four hours.

The patient was discharged from the hospital after the completion of treatment for his burn injury, without further complications from the incident, on preventive oral anticoagulation treatment for 12 months.

The incident was reported to the manufacturing company, and the catheter was sent for inspection. In the answering report, the manufacturer states that the incident was an isolated event resulting from a complex situation where the doctors took a calculated risk of leaving the catheter in place for a longer time than recommended. A complete examination of the catheter was not possible due to the condition of the catheter after the manipulations.

## 3. Discussion

Patients with severe burn injuries are highly susceptible to hypothermia due to multiple contributing factors, including extensive heat loss through damaged skin, administration of large fluid volumes during resuscitation, prolonged exposure during transport and wound management, and repeated operative procedures for burn excision and skin grafting [5,6,10]. Core body temperature may continue to decline during the initial hours following hospital admission, and restoration of normothermia may require several days [5].

Hypothermia is associated with significant physiological disturbances in burn patients, as it negatively affects immune function and stress responses, impairs platelet activity and coagulation pathways, and disrupts neurological and cardiovascular system homeostasis [6,10]. Additionally, it hinders surgical management of burn patients, mainly due to increased intraoperative bleeding and transfusion requirements, and a higher risk of postoperative infections. Often, early termination of operative procedures is imposed, resulting in reduced burn wound excision and grafting per session [10,11]. The relationship between hypothermia and mortality in burn patients remains controversial. While some studies suggest its association with worse outcomes, it has been debated whether hypothermia constitutes an independent predictor of mortality. Hostler et al. (2013) analyzed data from a national trauma registry, including 17,098 burn patients, and demonstrated that hypothermia was independently associated with increased mortality after adjusting for confounding variables, such as age, sex, and injury severity [5,12].

Given these serious consequences, prevention and rapid correction of hypothermia are essential but often challenging. Current warming strategies, such as raising ambient room temperature, administering warmed intravenous fluids, and utilizing forced-air warming systems, such as the Bair Hugger™, either alone or in combination, are frequently insufficient to maintain normothermia in patients with extensive burns [10]. Although elevating ambient temperature can reduce the hypermetabolic response and energy expenditure, it also presents disadvantages, including increased microbial growth and a physically stressful working environment for healthcare personnel [1,6,11].

Active internal temperature management has therefore been explored as an alternative approach. IVTM systems, initially introduced in the early 2000s for therapeutic hypothermia in patients with neurological injuries and post-cardiac arrest, have subsequently been applied for the treatment of hypothermia in burn patients. Corallo et al. (2007) [6] and Prunet et al. (2009) [13] were among the first to report the use of intravascular warming catheters (IVWC) in this population. There are studies suggesting that IVWC use may be more effective in maintaining normothermia during burn surgery compared to conventional warming techniques [6,11]. Davis et al. (2013) conducted a retrospective case–control study in patients with major burns undergoing surgery and reported that IVWC use reliably maintained intraoperative core temperature, potentially offering superior temperature control compared to traditional warming methods [11]. Similarly, Brown et al. (2024), in a prospective single-centre evaluation of the Thermogard XP^®^ system in critically injured burn patients, concluded that the device was effective in achieving and maintaining normothermia in patients with temperature dysregulation in the ICU and during surgery [7].

As with any invasive medical device, safety considerations are of paramount importance. CVC insertion is associated with risks related to placement, maintenance, and removal, including bleeding, arterial puncture, injury to adjacent structures, infection, catheter-related thrombosis, and catheter fracture or embolization [9].

While IVWC shares these general risks, concerns have been raised regarding potential device-specific complications, particularly related to the thrombogenicity of the balloon components. Although the increased catheter diameter and localized warming theoretically could increase thrombus formation, current evidence does not demonstrate a significantly higher complication rate compared with standard central venous catheters [9,10,11,13]. Reid et al. (2023) evaluated the incidence of venous thromboembolism (VTE) in patients managed with IVWC and reported a higher overall VTE incidence in the IVWC group (20%) compared to controls; however, these patients also exhibited greater illness severity and additional predisposing factors [14]. Importantly, only 10% of VTE events could be anatomically attributed to the catheter itself, a rate comparable to that observed with standard femoral CVCs in critically ill patients [14]. Regarding catheter dwell time, both clinical consensus and manufacturer guidelines recommend prompt removal of the IVWC once it is no longer required, to minimize the risk of complications [9,13,14].

In our patient, an ICY™ IVWC with three inflatable balloons was utilized. Although the recommended maximum duration of use is four days, the catheter was left in situ for eight days, due to the complexity of the patient’s clinical condition. Despite the prolonged dwell time, no evidence of deep vein thrombosis or insertion-site infection was detected on ultrasound and angiographic evaluation. The complication occurred during catheter removal. Catheter shearing or fracture is a recognized complication of central venous access and may result in difficulty during removal (stuck catheter). While excessive traction is generally discouraged, slow and gentle traction has been reported with some success. In cases where removal is unsuccessful, cutaneous cut-down with distal venotomy has been described as a safe and effective bedside technique [8].

To our knowledge, this represents the first published case of IVWC ICY™ balloon tearing resulting in impaired catheter removal and the need for interventional radiology techniques. According to the manufacturer’s operation manual, disruptions in the closed circuit are promptly detected, flow is interrupted, and alarm systems are activated. In the event of balloon rupture, a small volume of sterile saline may enter the circulation [9]. While not described in peer-reviewed publications, the FDA’s Manufacturer and User Facility Device Experience (MAUDE) database contains numerous reports of IVWC balloon failure, with saline leakage into the circulation, with no deleterious effects for the patients, including a case complicated by difficulty during catheter removal [15]. In the present case, although no device malfunction was detected prior to removal, mechanical detachment and folding of the middle balloon resulted in a sequence of events requiring targeted intervention, fortunately without long-term adverse sequelae for the patient. Clinicians should be aware of this previously unpublished complication of IVWC use. Careful handling can prevent more serious sequelae, such as embolization of fragmented catheter parts. Finally, although it remains unclear if the reported adverse event was related to prolonged use of the device, timely removal of intravascular devices, when no longer indicated, remains essential.

## 4. Conclusions

Patients with major burn injury are prone to hypothermia, potentially resulting in an increase in length of hospital stay and mortality. One of the major challenges faced is confronting their hypermetabolic state, and all measures available should be used to preserve body temperature. The intravascular temperature management system is a relatively new device carrying the inherent risks of central venous catheters, with no definitive evidence to indicate any difference in complication rates. We report an infrequent adverse event of the complicated removal of the ICY^TM^ catheter. Physicians who use these devices should be aware of this possible complication in order to be prepared for its management. In any case, to reduce risks, the catheter should be removed promptly when no longer needed, and the maximum use duration, recommended by the manufacturer, must be followed.

## Data Availability

The data presented in this study are available upon request from the corresponding author due to privacy restrictions.

## Abbreviations

The following abbreviations are used in this manuscript:

ICU	Intensive Care Unit
CVC	Central Venous Catheter
TBSA	Total Body Surface Area
BICU	Burns Intensive Care Unit
ABSI	Abbreviated Burn Severity Index Score
IVTM	Intravascular Temperature Management
IVWC	Intravascular Warming Catheters
VTE	Venous Thromboembolism
